# Novel biallelic variant in *BBS9* causative of Bardet–Biedl syndrome: expanding the spectrum of disease-causing genetic alterations

**DOI:** 10.1186/s12920-021-00943-w

**Published:** 2021-03-26

**Authors:** Julia Suárez-González, Verónica Seidel, Cristina Andrés-Zayas, Elvira Izquierdo, Ismael Buño

**Affiliations:** 1 Genomics Unit, Gregorio Marañón General University Hospital, Gregorio Marañón Health Research Institute (IiSGM), C/Doctor Esquerdo 46, 28007 Madrid, Spain; 2 Gregorio Marañón Health Research Institute (IiSGM), Madrid, Spain; 3 Clinical Genetics, Department of Pediatrics, Gregorio Marañón General University Hospital, Madrid, Spain; 4 Pediatric Nephrology, Department of Pediatrics, Gregorio Marañón General University Hospital, Madrid, Spain; 5 Department of Hematology, Gregorio Marañón General University Hospital, Madrid, Spain; 6 Department of Cell Biology, School of Medicine, Complutense University of Madrid, Madrid, Spain

**Keywords:** Bardet–Biedl syndrome, *BBS9*, Novel genomic variant, Splice-site, Pathogenic variants

## Abstract

**Background:**

Bardet–Biedl syndrome (BBS) is a rare autosomal recessive ciliopathy disorder. Many BBS disease-causing genetic variants have been identified due to the advancement of molecular diagnostic tools. We report on a novel pathogenic variant in a consanguineous Pakistani family with an affected child.

**Case presentation:**

Clinical exome sequencing was used to search for BBS causing variants in the affected individual and identified a novel homozygous splice-site variant in the *BBS9* gene (c.702 + 1del). Sanger sequencing was performed for variant validation and segregation studies. Expression analysis using mRNA levels to assess the functional impact of the novel variant demonstrated skipping of exon 7 in the affected alleles, suggesting a truncating effect. Three-dimensional structural modelling was used to predict pathogenicity of the variant residue and the alteration leads to a partial deletion of the PHTB1_N domain and a total deletion of the PHTB1_C domain.

**Conclusion:**

The study of this case expands the spectrum of biallelic variants in the *BBS9* gene associated with BBS and increased the knowledge on the molecular consequences of splicing variation c.702 + 1del.

**Supplementary Information:**

The online version contains supplementary material available at 10.1186/s12920-021-00943-w.

## Background

The Bardet–Biedl syndrome (BBS, MIM#209900) is a rare hereditary condition with an estimated prevalence that is higher in isolated or consanguineous populations (Bedouin, 1:13,500 newborns [1]; Arab, 1:65,000 newborns [2]) as compared to more varied populations in Europe or North America (1:160,000 to 1:140,000 newborns [3], respectively). BBS is a clinically heterogeneous autosomal recessive ciliopathy characterized by postaxial polydactyly, cystic nephropathy, retinal dystrophy, developmental delay/cognitive impairment, genital anomalies and truncal obesity, as primary features. Additional, secondary features include other neurological problems (ataxia, hypertonia), Hirschsprung disease, endocrine disturbances such as type 2 diabetes mellitus and hypercholesterolemia, liver involvement, dental anomalies, congenital heart disease, and anosmia/hyposmia. Four primary features or three primary features plus two secondary features are required for a clinical diagnosis of BBS [4].

BBS is also genetically heterogeneous and occurs as a result of defects in genes encoding many cilia-related proteins. Alterations in these genes lead to ciliary dysgenesis and dysfunction. Up to three pathogenic variant alleles may be required for clinical manifestation of some forms of the disease [5]. To date, 20 genes causing BBS have been identified: *BBS1, BBS2, ARL6, BBS4, BBS5, MKKS, BBS7, TTC8, BBS9, BBS10, TRIM32, BBS12, MKS1, CEP290, WDPCP, SDCCAG8, LZTFL1, BBIP1, IFT27 and AZI1*; pathogenic variants in which explain approximately 80% of patients with BBS [4]. Genotype–phenotype correlations are unclear, and great clinical variability is observed within and between families [6].

The present report describes a Pakistani consanguineous family with an affected child presenting with clinical features of BBS. Molecular analysis revealed a homozygous novel RNA-splicing alteration in the *BBS9* gene and allowed to identify the contribution of this splicing variant encoding for an alternative parathyroid hormone-responsive B1 (PTHB1) protein.

## Case presentation

The proband was a 12-year-old boy, the second child of four born to a consanguineous couple from Pakistan, who was referred to the Genetics clinic by the neuropediatrician who had been following him for intellectual disability. His past medical history included global developmental delay (he started gait and speech at age 4 years), visual impairment with night blindness since age 6 years and overweight since age 3–4 years. His perinatal history was unremarkable except for postaxial polydactyly of both hands and feet. The extra digits of the hands had been removed and surgery on the feet was planned due to pain and walking difficulties. On clinical examination his height was 145.5 cm (− 0.6 SD), weight 64 kg (1.6 SD), head circumference 50.3 (− 2.1 SD) and body mass index 30.23 kg/m^2^ (4.2 SD on World Health Organization charts), indicating overweight and microcephaly. He had nystagmus, brachydactyly, broad hands and feet, small penis and no pubertal changes. Examination of his fundus showed the features of retinitis pigmentosa and neurophysiological exams found bilateral absence of visual potentials and retinal responses. A renal evaluation showed normally functioning kidneys and no anomalies on ultrasound scan. Altogether the clinical data led to a diagnosis of BBS. Blood samples for molecular genetic testing were collected upon written informed consent using protocols approved by an institutional review board in compliance with the principles of the Declaration of Helsinki.

Blood samples were obtained from the affected individual and from his unaffected parents. DNA was isolated from 2 ml of blood using the DNA Blood Mini kit (QIAGEN, Germany) according to the manufacturer’s specifications. Clinical exome sequencing and further genetics analysis are described in Additional file 1.

**Fig. 1:**
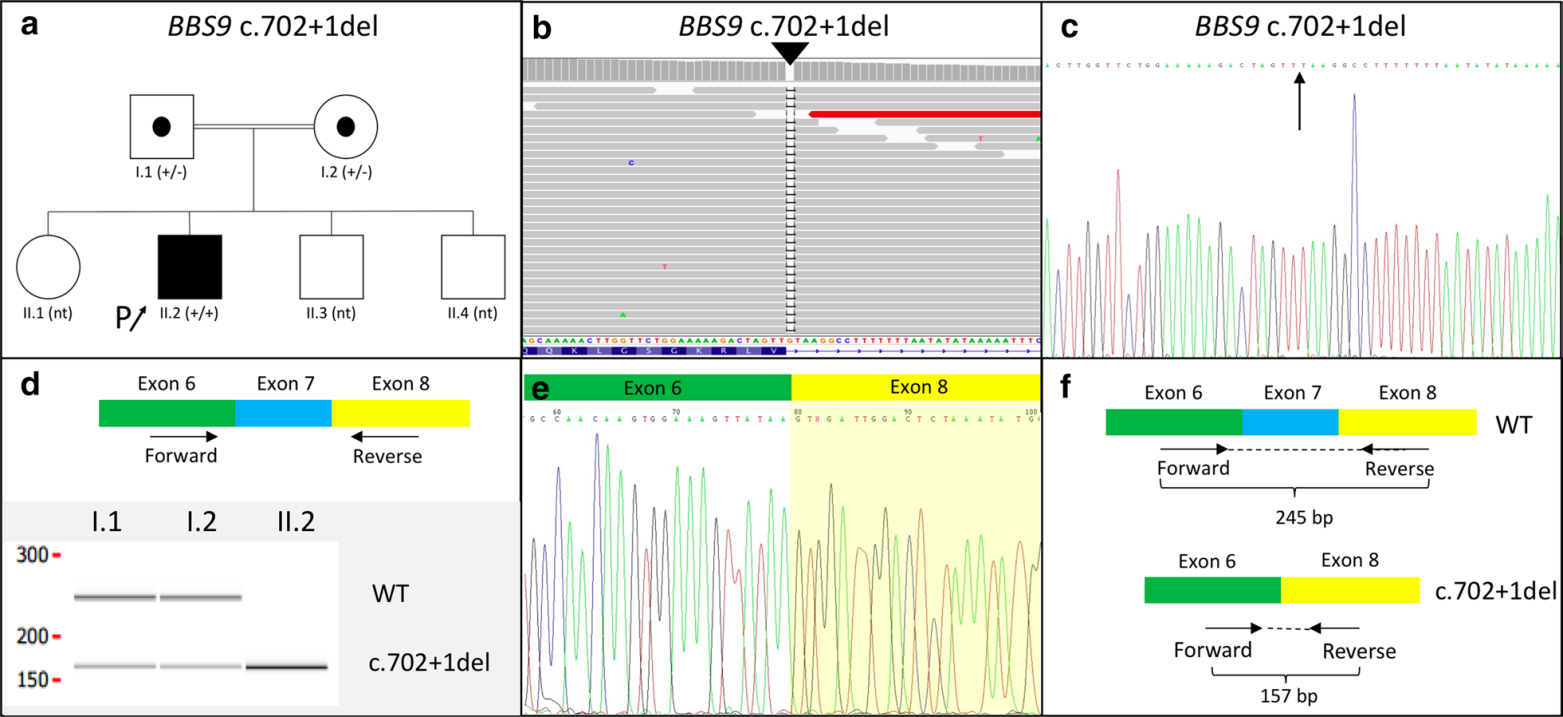
**a** Pedigree of the family showing the affected proband (arrow), homozygous for the c.702 + 1del variant in the *BBS9* gene, and his heterozygous (carrier) parents. The three siblings of the proband have not been tested (nt: not tested). **b**, **c** DNA sequencing of proband with NGS (**b**) and subsequent validation with Sanger (**c**) showing the novel gene variation identified, c.702 + 1del variant affecting the first base of intron 7–8 (arrow). **d** Microfluidic technology after RT-PCR using primers located in exons 6 and 8. A band of 245 bp corresponding to the wild-type (Wt) gene and a band of 157 bp corresponding to the variant lacking exon 7 is shown in the heterozygous parents (I.1 and I.2) while only the variant form is seen in the homozygous proband (II.2). **e** Sanger sequencing of the splicing variant resulting from the skipping of the whole exon 7. **f** Interpretation of the empirical observations combining the results of RT-PCR and Sanger sequencing from both Wt and c.702 + 1del alelles

The sequencing of 4572 genes led to identified a homozygous pathogenic variant in the *BBS9* gene (ENSG00000122507): c.702 + 1del (Fig. 1a–c; Additional file 2). Segregation analysis revealed that both parents were heterozygous carriers for the variant (Fig. 1a).

The HSF [7] tool showed that the score of the c.702 + 1del variant (mutant score 18.44) was significantly decreased as compared to the wild-type (score 80.69). We therefore suspected that the altered sequence may break the original splice site which is predicted to be a donor splicing variant, and consequently affect pre-mRNA processing. These results prompted us to evaluate the impact of the variant at the RNA level in order to elucidate a possible effect on splicing.

**Fig. 2:**
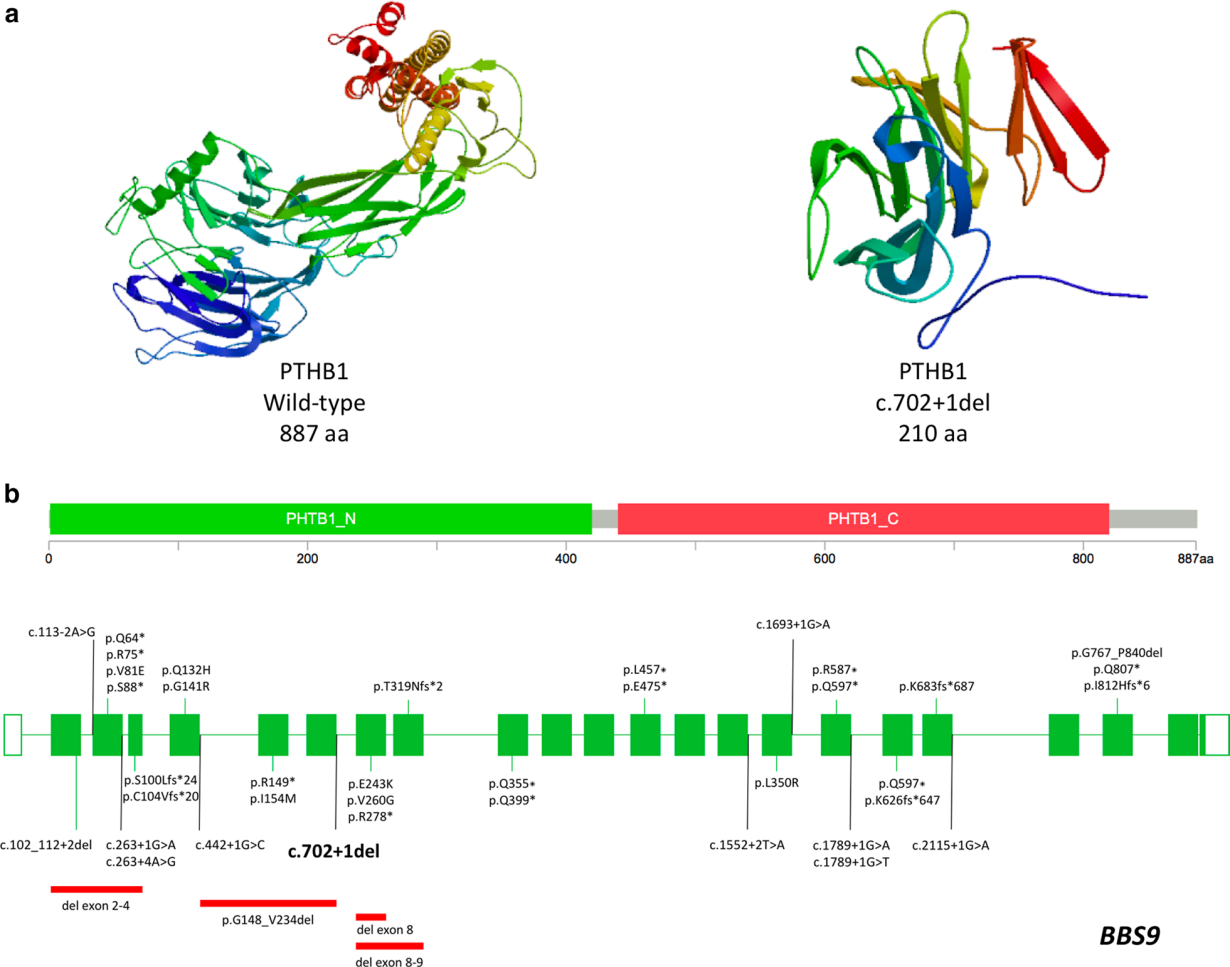
**a** Three-dimensional structures of wild-type and altered PTHB1 proteins. **b** Spectrum of *BBS9* pathogenic variants in Bardet–Biedl syndrome. Coding exons are depicted as green boxes, non-coding exons are depicted as white boxes, green arrows indicate variants in coding regions, black arrows indicate splice-site variants and red lines indicate gross deletions (ENST00000242067.11)

Reverse transcription (RT) PCR reactions were performed after cDNA synthesis with a random hexamer-primer based kit (Transcriptor First Strand cDNA synthesis, Roche) using a primer pair spanning exons 6–8 of the *BBS9* gene expected to amplify a 245-bp fragment (Fig. 1d). The size of RT-PCR products was established by microfluidic LabChip technology (Perkin Elmer, USA), and direct Sanger sequencing of the products was performed using standard procedures (Fig. 1d–f, Additional file 1). Besides the expected 245-bp PCR product, a smaller fragment of 157-bp, with an 88-bp deletion, was amplified in both parents of the proband (heterozygous carriers). The affected individual only showed the smaller fragment (homozygous for the deletion; Fig. 1d). Sequencing of the smaller fragment to elucidate the nature of the missing sequence (Fig. 1e) revealed a transcript lacking the whole exon 7 (Fig. 1e, d). Additionally, detailed sequence analysis shows that such deletion results in a frameshift alteration leading to an introduction of a premature stop codon. Subsequent in silico analysis [8, 9] showed that the c.702 + 1del variant leads to a truncated protein of 210 amino acids, in comparison to the wild-type protein of 887 amino acids (UnitProt: Q3SYG4; Fig. 2). The truncated protein has a partial deletion of the PHTB1_N domain and a total deletion of the PHTB1_C domain, as determined by three-dimensional modelling (Fig. 2a).

Mapping the positions of the pathogenic variants reported in the *BBS9* gene revealed a non-uniform distribution. Most nucleotide changes lead to nonsense variants (34%, 13/38) and about 29% (11/38) of all variants occur at intron–exon boundaries and affect splicing donor or acceptor sites (Fig. 2b) [1, 10–20].

## Discussion and conclusions

Since the first description of the BBS, a pleiotropic autosomal recessive disorder, there has been extensive development in the understanding of the molecular basis of BBS [21]. BBS is a genetic disorder with at least 20 known causative genes, all of which code for proteins involved in the development and function of primary cilia. Primary cilia are specialized organelles located at the surface of almost every vertebrate cell and they are involved in the regulation of key signal transduction pathways. Perturbation of ciliary proteins can give rise to a broad range of phenotypes in mammals, called ciliopathies [6]. *BBS9* gene encodes for 1 of the 7 BBS highly conserved proteins that form the stable core of this BBS protein complex, required for ciliogenesis [20]. BBS patients are typically affected with obesity, retinal degeneration, kidney anomalies, polydactyly and olfactory deficits. Due to the noteworthy interfamilial and intrafamilial variability in clinical presentation, early genetic testing can be conducted to confirm the diagnosis and provide genetic counselling to the families. Nowadays, the analysis of a multigene panel by NGS provides the most effective approach in achieving a molecular diagnosis of BBS.

In the present report, a novel pathogenic splice site variant in the 7–8 intron of the *BBS9* gene (c.702 + 1del) was identified in a patient from a consanguineous Pakistani family whose clinical spectrum was consistent with BBS. In general, variants in the canonical acceptor and donor splicing sites affect highly conserved sequences that define exon–intron boundaries. These variants might alter the interaction between pre-mRNA and the proteins involved in intron removal processes. In this case, experimental evidence by cDNA analysis of exon 6 and 8 of the *BBS9* gene confirmed the prediction of pathogenicity of this novel variant (Fig. 1d–f).

This novel variant caused aberrant splicing resulting in whole exon 7 skipping, which leads to a truncated protein with partial loss of the PHTB1_N domain and total deletion of the PHTB1_C domain of BBS9 (Fig. 2a).

This particular variant has not been reported yet in the medical literature. We also collected the pathogenic and probably pathogenic variants in *BBS9* gene reported in human genetics variations databases. The main alterations observed are nonsense variants along the gene (34%). Additionally, frameshift, splice site variants and large deletions, all leading to a truncated protein, summarize up to 80% of all known variants. Consequently, the main disease causing mechanism may be a loss of function of the BBS9 protein (Fig. 2b). Splice site variants in *BBS9* are known to be pathogenic and they have been described in several families with BBS (Fig. 2b) [11, 20]. However, there is still no established way to predict how these variants will affect the manner in which the pre-mRNA is spliced. Our in vitro experiment yielded an empirical result fully compatible with the theory of exon skipping. Hence, although we cannot directly confirm that the novel variant reported here had influence on the phenotype of this case, it increases the genomic variant spectrum of the *BBS9* gene.

Although there have been associations between certain genes and phenotypic features, genotype–phenotype correlations of *BBS9*-related patients are still poor and additional studies are needed to better characterize the disease.

## Supplementary Information

**Additional file 1:** Supplementary material of the DNA, RNA and in silico analysis.

**Additional file 2:** All variants detected in the clinical exome sequencing. Dataset analized will be accessible with the following link: https://www.ncbi.nlm.nih.gov/sra/PRJNA706860.

## Data Availability

The datasets analysed during the current study are deposited in NCBI sequence Read Archive (SRA). Records will be accessible with the following link: https://www.ncbi.nlm.nih.gov/sra/PRJNA706860. The accession numbers corresponding to the reference sequences used in this study can be found in Additional file 2 and can be obtained from the NCBI Nucleotide database. Reference sequences used in this study for *BBS9* analysis can be found in Ensembl database with the following links: https://www.ensembl.org/Homo_sapiens/Gene/Summary?db=core;g=ENSG00000122507;r=7:33109557-33877180. https://www.ensembl.org/Homo_sapiens/Transcript/Summary?db=core;g=ENSG00000122507;r=7:33129564-33606069;t=ENST00000242067. Public databases used in this study included human hg19 reference genome assembly (http://hgdownload.soe.ucsc.edu/goldenPath/hg19/bigZips/hg19.fa.gz).

## Abbreviations

BBS: Bardet–Biedl syndrome
PTHB1: Parathyroid hormone-responsive B1
