# Supplementary material for: Novel biallelic variant in BBS9 causative of Bardet–Biedl syndrome: expanding the spectrum of disease-causing genetic alterations
Source: BMC Med Genomics. 2021 Mar 26;14:91. doi: 10.1186/s12920-021-00943-w (PMC7995718; doi:10.1186/s12920-021-00943-w)
Supplement: Supplementary file 1 — Additional file 1: Supplementary material of the DNA, RNA and in silico analysis. [file 12920_2021_943_MOESM1_ESM.docx]

**ADDITIONAL FILE 1**

**Novel biallelic variant in *BBS9* causative of Bardet-Biedl syndrome. Expanding the spectrum of disease-causing genetic alterations.**

Julia Suárez-González^1,2^, Veronica Seidel^3#^, Cristina Andrés-Zayas^1,2#^, Elvira Izquierdo^4^, Ismael Buño^1,2,5,6,*^

^1^Genomics Unit, Gregorio Marañón General University Hospital, Gregorio Marañón Health Research Institute (IiSGM), Madrid, Spain.

^2^Gregorio Marañón Health Research Institute (IiSGM), Madrid, Spain.

^3^Clinical Genetics, Department of Pediatrics, Gregorio Marañón General University Hospital, Madrid, Spain.

^4^Pediatric Nephrology, Department of Pediatrics, Gregorio Marañón General University Hospital, Madrid, Spain.

^5^Department of Hematology, Gregorio Marañón General University Hospital, Madrid, Spain.

^6^Department of Cell Biology, School of Medicine, Complutense University of Madrid, Spain.

(#) VS and CA-Z contributed equally to this work.

^*^Corresponding Author:

Ismael Buño, PhD

Genomics Unit

Gregorio Marañón General University Hospital

Gregorio Marañón Health Research Institute (IiSGM)

C/ Doctor Esquerdo 46

28007 Madrid

TEL: 915860151

E-mail: ismaelbuno@gmail.com

**ADDITIONAL FILE 1**

**DNA sequence analysis**

Clinical exome (Sophia Genetics, Switzerland) was performed in proband. A total of 21,191,698 reads were produced on NextSeq 500 platform (Illumina lnc., USA) using NextSeq Mid-Output Reagent Kit (2x150 cycles). Reads were aligned to the hg19 genomic sequence.

Sequencing data were analysed using SOPHIA DDM variant analysis software (Sophia Genetics, Switzerland) and Integrative Genomics Viewer (Broad Institute, USA). Variants identified by clinical exome sequencing were filtered by consequence (Non-synonymous variants located in coding or splicing regions of canonical isoforms) and allele frequency less than 0,1% in public databases (1000 Genomes Project, Exome Aggregation Consortium, GnomAD) and frequency less than 100 in Sophia Genetic Database. The Human Gene Mutation Database (HGMD) and ClinVar database were used to screen for previously reported pathogenic or probably pathogenic variants (Appendix S2). The pathogenicity of variants was designated according to the recommendation of the American College of Medical Genetics and Genomics.

The pathogenic variant detected by next generation sequencing (NGS) analysis was validated through Sanger sequencing (BBS9.Exon7.Fw: 5´-AAGGCAGGAGACTGAACAGC-3´ and BBS9.Exon7.Rv: 5´-CATGACCAGGCATGTGTTTC-3´) on an ABI 3130xl Genetic analyser (Applied Biosystems, USA) using Big Dye Terminator cycle sequencing v3.1 ready reaction Kit (Life Technologies, USA; Reference sequence: ENST00000242067.11). Pathogenic variant found in the proband was then analysed in the progenitors using Sanger sequencing.

Table 1. Clinical exome sequencing statistics

| Total reads | 21,191,698 |
| --- | --- |
| Mapped reads | 20,407,258 |
| % Mapped reads | 96,3% |
| % Bases on target | 95,36% |
| Mean coverage | 81 |
| % target bases covered ≥ 25x | 99,6% |
| % target bases covered ≥ 50x | 97,6% |
| % target bases covered ≥ 100x | 82,1% |
| Coverage heterogeneity | 0,76% |

**Effect of the variant: *In-silico* analysis**

The Human Splicing Finder v3.0 (HSF) algorithms were employed to predict the possible influence of the variant on pre-mRNA splicing.^6^ Translation of nucleotide sequence to a protein sequence was performed with the Translate tool of ExPASy bioinformatics resource.^7^ A three-dimensional model was established to predict the probable conformational structure of the altered protein using the SWISS-MODEL workspace.^8^

**Effect of the variant: RNA analysis**

To evaluate the transcript variants of *BBS9* by RT-PCR in the blood cells from the patient and his parents, total RNA was isolated using Trizol and phenol-chloroform extraction. cDNA synthesis was performed using Transcriptor First Strand cDNA synthesis (Roche, Switzerland). cDNA analysis was carried out by PCR amplification using specific primers (Exon6.Fw: 5´-CCTGGTCCTCTTGCCTACAG-3´ and Exon8.Rv: 5´-AAAACAGAGGATGCCGACTG-3´). The end-point PCR amplification products were analysed by microfluidic using the LabChip instrument (Perkin Elmer, USA), and their identity was confirmed by Sanger sequencing.

**Disease-causing variant** **spectrum of *BBS9***

All pathogenic and probably pathogenic variants associated with BBS that occur in the *BBS9* gene and are described in the Human Gene Mutation Database and ClinVar, were reviewed.
